# A prognostic signature consisting of metabolism-related genes and SLC17A4 serves as a potential biomarker of immunotherapeutic prediction in prostate cancer

**DOI:** 10.3389/fimmu.2022.982628

**Published:** 2022-10-17

**Authors:** He Li, Jie Gu, Yuqiu Tian, Shuyu Li, Hao Zhang, Ziyu Dai, Zeyu Wang, Nan Zhang, Renjun Peng

**Affiliations:** ^1^ The Animal Laboratory Center, Hunan Cancer Hospital and The Affiliated Cancer Hospital of Xiangya School of Medicine, Central South University, Changsha, Hunan, China; ^2^ Department of Geriatric Urology, Xiangya International Medical Center, Xiangya Hospital, Central South University, Changsha, Hunan, China; ^3^ National Clinical Research Center for Geriatric Disorders, Xiangya Hospital, Central South University, Changsha, China; ^4^ Martini-Klinik Prostate Cancer Center, University Hospital Hamburg-Eppendorf, Hamburg, Germany; ^5^ Department of Infectious Disease, Zhuzhou Central Hospital, Zhuzhou, Hunan, China; ^6^ Department of Thyroid and Breast Surgery, Tongji Hospital, Tongji Medical College of Huazhong University of Science and Technology, Wuhan, Hubei, China; ^7^ Department of Neurosurgery, Xiangya Hospital, Central South University, Changsha, Hunan, China; ^8^ One‑Third Lab, College of Bioinformatics Science and Technology, Harbin Medical University, Harbin, Hei Longjiang, China

**Keywords:** prostate cancer, metabolism, prognostic model, immuno-/chemotherapy response, immune infiltration

## Abstract

**Background:**

Prostate cancer (PCa), a prevalent malignant cancer in males worldwide, screening for patients might benefit more from immuno-/chemo-therapy remained inadequate and challenging due to the heterogeneity of PCa patients. Thus, the study aimed to explore the metabolic (Meta) characteristics and develop a metabolism-based signature to predict the prognosis and immuno-/chemo-therapy response for PCa patients.

**Methods:**

Differentially expressed genes were screened among 2577 metabolism-associated genes. Univariate Cox analysis and random forest algorithms was used for features screening. Multivariate Cox regression analysis was conducted to construct a prognostic Meta-model based on all combinations of metabolism-related features. Then the correlation between MetaScore and tumor was deeply explored from prognostic, genomic variant, functional and immunological perspectives, and chemo-/immuno-therapy response. Multiple algorithms were applied to estimate the immunotherapeutic responses of two MeteScore groups. Further *in vitro* functional experiments were performed using PCa cells to validate the association between the expression of hub gene SLC17A4 which is one of the model component genes and tumor progression. GDSC database was employed to determine the sensitivity of chemotherapy drugs.

**Results:**

Two metabolism-related clusters presented different features in overall survival (OS). A metabolic model was developed weighted by the estimated regression coefficients in the multivariate Cox regression analysis (0.5154*GAS2 + 0.395*SLC17A4 - 0.1211*NTM + 0.2939*GC). This Meta-scoring system highlights the relationship between the metabolic profiles and genomic alterations, gene pathways, functional annotation, and tumor microenvironment including stromal, immune cells, and immune checkpoint in PCa. Low MetaScore is correlated with increased mutation burden and microsatellite instability, indicating a superior response to immunotherapy. Several medications that might improve patients` prognosis in the MetaScore group were identified. Additionally, our cellular experiments suggested knock-down of SLC17A4 contributes to inhibiting invasion, colony formation, and proliferation in PCa cells *in vitro*.

**Conclusions:**

Our study supports the metabolism-based four-gene signature as a novel and robust model for predicting prognosis, and chemo-/immuno-therapy response in PCa patients. The potential mechanisms for metabolism-associated genes in PCa oncogenesis and progression were further determined.

## Introduction

Prostate cancer (PCa) accounts for one of the most prevalent malignancies among males worldwide and ranks the second highest cause of elderly male tumor-related deaths (1, 2). Most patients with localized PCa receive standard therapy including androgen deprivation therapy, radical prostatectomy or radiotherapy, which leads to favorable cancer control (3, 4). However, approximately 20-30% of patients will develop a castration-resistant or biochemical recurrence (BCR), and such patients are more likely to suffer metastases and cancer-specific mortality (5). Therefore, exploring the tumor biomarker model that can classify the subtypes of PCa and predictively determines efficacious risk signatures remains crucial. Over the past few decades, clinical-stage, prostate-specific antigens (PSA) and Gleason scores were mainly employed to diagnose and monitor the prognosis of PCa patients (6, 7). These clinic pathological parameters do not possess favorable specificity and sensitivity in assessing the prognosis of PCa patients (8, 9). Particularly, PSA has undergone some discredit for it might bring overdiagnosis and overtreatment in adequately treated patients through active surveillance (9). In this aspect, better informative biomarkers are desperately required to evaluate the increased risk of overall survival (OS).

PCa exhibits distinct statuses of metabolism from normal tissues thereby supplying a novel approach to distinguish tumors *via* metabolic differences. Recent research interpreted that specific metabolisms are closely associated with PCa, for instance, citrate, lipid and choline (10). A study reported urea cycle metabolites increased in PCa utilizing capillary electrophoresis and mass spectrometry (11). Studies demonstrated that PCa cells consume large amounts of glucose during the metastatic stage (12, 13) and highly glycolytic metabolism PCa patients showed a poor prognosis (14). In addition, activation of glycolysis leads to increased generation of lactic acid that facilitates several tumor-accelerating procedures, such as stemness properties, cancer invasion and metastasis, angiogenesis, inhibition of antitumor immune response, and hypoxia resistance (15, 16). However, the correlation between the metabolism gene signature and OS of PCa is yet poorly defined. An understanding of the cellular metabolism of PCa is essential in the prediction of prognosis and development of potential metabolically targeted treatments.

Here, we developed a metabolism-based four-gene model for predicting prognosis and chemo-/immuno-therapy response utilizing The Cancer Genome Atlas (TCGA-PRAD) and Gene Expression Omnibus (GEO) dataset (GSE16560). Furthermore, we performed cellular experiments to explore the correlation between the SLC17A4 expression and the *in vitro* proliferation and invasion phenotype of PCa cells.

## Materials and methods

### Data collection and preprocessing

We collected PCa gene expression data from several publicly available databases. A total of 777 samples from PCa patients were enrolled in the study: 496 from TCGA-PRAD (training cohort) and 281 from GSE16560 (validation cohort). The clinical and RNA-sequencing (RNA-seq) data were obtained from The Cancer Genome Atlas (TCGA, http://cancergenome.nih.gov). The GSE16560 datasets were derived from the Gene Expression Omnibus (GEO, https://www.ncbi.nlm.nih.gov/geo/).

### Identification of PCa MetaCluster

A published list of 2577 metabolism-associated genes was acquired for subsequent clustering (17). Univariate Cox was employed to filter the candidate genes (p< 0.01) and 46 candidate metabolic genes were screened for clustering in the TCGA-PRAD cohort. PCa patients with distinct metabolic gene profiles were stratified utilizing k-means algorithm, which confirmed metabolic-associated patterns and classified patients for further evaluation using the R “ConsensusClusterPlus” package (18). Afterward, the correlation between the MetaCluster and the collected 114 metabolic pathways was calculated (19).

### Establishment of metabolic gene signature

The R “limma” package was adopted to identify differentially expressed genes |(log FC)| > log2(1.5) & P < 0.05 was selected for analysis (20). Subsequently, univariate Cox regression was performed to determine prognostic MetaGenes (P< 0.01). We then applied random survival forest algorithm through the R “randomForestSRC” package to screen out the more valuable MetaGenes with prognostic potential (variable relative importance> 0.3) (21). The metabolic gene signature was constructed based on all different combinations of prognostic MetaGenes and weighted by their estimated regression coefficients in multivariate Cox regression analysis. The final metabolic gene signature named MetaScore was identified with the highest 5 years-area under the curve (AUC). The classification was conducted with model-based hierarchical agglomerative clustering based on the Gaussian finite mixture model (GMM) (22).

### Validating the accuracy of the MetaScore

The MetaScore of the 496 PCa patients in the TCGA-PRAD dataset was estimated, and then we stratified the PCa patients into high- and low-MetaScore groups according to the best cutoff. The Kaplan-Meier curve analyzed the associations between OS and MetaScore. TimeROC was employed to verify the efficiency and accuracy of the prognosis predictions of MetaScore for 1-, 3- and 5-year. Univariate and multivariate cox regression analysis was employed on the MetaScore and individual clinical variables including age and tumor stage of patients (T and N).

### Analysis of pathway enrichment and functional annotation

Gene Ontology (GO) and Kyoto Encyclopedia of Genes and Genomes (KEGG) related gene sets were obtained from the MSigDB (23). Gene set enrichment analysis (GSEA) was conducted using the R “clusterProfiler” package (24) and gene set variation analysis (GSVA) was performed through the R “GSVA” package (25).

### Genomic alteration characteristics

We utilized GISTIC 2.0 (http://www.broadinstitute.org/cancer/software/genepattern) to explore the somatic copy number alternations (SCNAs) in PCa based on TCGA-PRAD. Patients were categorized into low- and high-MetaScore groups. The specific genomic enrichment, copy number alternations (CNAs), and the threshold copy number (CN) at alteration peaks related to MetaScore were detected. We used “maftools” R package for the analysis of somatic mutations. Subsequently, tumor mutation burden (TMB) (26) was calculated based on the TGCA-cohort somatic mutations to evaluate the mutation status between different MetaScore groups.

### Estimation of immune infiltration and immune checkpoint

R “IOBR” package was used for immune infiltration assessment (27). CIBERSORT algorithm (28), MCPcounter algorithm (29), ssGSEA algorithm (30), and TIMER algorithm (31) were employed to evaluate the relative fraction of the immune cell in the TCGA-PRAD cohort. ESTIMATE algorithm was applied for calculating the ESTIMATE score and tumor purity (32). The correlations between MetaScore groups and immune checkpoint expression were analyzed.

### Prediction of immunotherapy for PCa patients

The IMvigor dataset was downloaded from a freely available database, which included installed software and R “IMvigor210CoreBiologies” package (http://research-pub.gene.com/IMvigor210CoreBiologies). Immunotherapy predictive value of the four-gene model was verified in multi-datasets [GSE35640 (anti-recMAGE A3, metastatic melanoma and non-small-cell lung cancer), GSE78220 (anti-PD-1, melanomas), and GSE91061 (anti-CTLA4 and ant-PD1, advanced melanoma)]. Survival probability for PCa patients with high- and low-MetaScore in the IMvigor and GSE78220 cohort was investigated. Wilcoxon test was performed in Microsatellite instability (MSI) (33) to describe the differences in immunotherapeutic response between MetaScore groups.

### Prediction of chemotherapy response

The chemotherapeutic response in PCa patients was assessed using the Genomics of Drug Sensitivity in Cancer database (GDSC, https://www.cancerrxgene.org) by R “oncoPredict” package (34). PCa patients’ drug sensitivity in PRISM and CTRP2.0 was measured by R “pRRophetic” package (35).

### Cell culture and transfection

The prostate cancer cell lines PC3 and DU145 were employed to explore the effect of SLC17A4 on PCa. PC3 cells and DU145 cells were cultured in RPMI 1640 medium (Biological Industries) with 10% fetal bovine serum (FBS) and 1% penicillin-streptomycin (Beyotime Biotechnology, China). Cultures were done in a 37°C humidified incubator with 5% CO_2_. The cells were digested and passed with a ratio of 1:6 upon attaining 80% density. Each experiment was performed in triplicate. Specific siRNAs targeting SLC17A4 were designed and synthesized from Sangon Biotech (Shanghai, China). The transfection was conducted using Lipofectamine 2000 (Invitrogen, USA) according to the manufacturer’s instructions. Briefly, cells were transfected in 24-well plate in a total amount of 5 μl (20 μM) siRNA-NC or siRNA-SLC17A4 (si-RNA-626 or si-RNA-1080) with 5 μl of lipofectamine 2000. Then, the medium was changed after 6 h of transfection and samples were collected for subsequent assays after 48 h incubation.

### RNA isolation and RT-qPCR

The total mRNA was extracted from transfected cells by the TRIzol solution (Thermo Fisher, USA). The mRNA reverse transcription kit was purchased from Cwbio (China) for reverse-transcription of mRNA to cDNA. The primers sequences of GAPDH were ACAGCCTCAAGATCATCAGC (Forward), GGTCATGAGTCCTTCCACGAT (Reverse). The primers of SLC17A4 were GCACTCTTCCTCCCTCAGTA (Forward), ATTCATCCACTATCCCTTTCCTG (Reverse). The cycling conditions were as follows: 95° for 10 min, followed by 40 cycles at 95° C for 15 s and 60° C for 30 s. GAPDH was employed as an internal control to normalize the relative mRNA expression levels.

### Antibodies and western blot

The total protein concentration was quantified by the BCA method. Proteins were separated by 10% SDS-PAGE and then they were transferred from the gel to an NC membrane. The membrane was incubated overnight at 4 °C with primary antibody SLC17A4 (0.5µg/ml, Thermo Fisher, USA) and β-actin (1: 5000, ProteinTech, USA) after being blocked for 1.5 h. Signals were detected by ECL reagent after incubation with the corresponding secondary antibody.

### CCK-8 assay

The Cell Counting Kit-8 (DOJINDO, Japan) was employed to evaluate cell proliferation. The transfected PC3 and DU145 cells with 1000 cells/well were inoculated in 96-well plates. CCK-8 solution (10 μl) was added to each well, and cell proliferation was measured at 24, 48, and 72 h.

### 5-ethynyl-2’-deoxyuridine assays

The transfected PC3 and DU145 cells were plated into a 20 mm round coverslip. The operations were performed following the instruction manual using the EdU Cell Proliferation Assay Kit (Ribobio, China).

### Transwell assay

The upper chambers of the Transwell contain a membrane (8-μm pore; Corning, USA) that was placed into 6-well plates. Next, the upper chamber was inoculated with 100 μl cell suspensions (2*10^6^ cells/ml) maintained in a serum-free medium, and the lower chamber was filled with a 500 μl culture medium supplemented with 10% FBS. After 48 h of culture, the invasion cells were fixed (4% Paraformaldehyde) and stained (0.1% Crystal Violet).

### Colony formation assay

The transfected PC3 and DU145 cells were seeded into 6-well plates (200 cells/well) and then incubated for 2 weeks. The colonies were then fixed for 15 min with 4% Paraformaldehyde solution (1 ml/well) and stained for 30 min with crystal violet reagent (Solarbio, China). The stained colonies were photographed and computed.

### Statistical analysis

The Shapiro–Wilk test was employed to detect whether the variables were normally distributed. The Wilcoxon test and Kruskal-Wallis test was utilized to compare the non-normally distributed data between the two groups and multiple groups, respectively. Unpaired Student’s t-test and one-way analysis of variance (ANOVA) was used to compare normally distributed variables between the two groups and multiple groups, respectively. The Kaplan-Meier survival plots were used to estimate OS between two groups using the R package “survminer”. The Cox regression for survival analysis was performed by R package “survival”. Time-dependent receiver operating characteristic (ROC) curves were plotted using the R package “timeROC”. All heatmaps were conducted through R “ComplexHeatmap” package. The data were mainly visualized using ggplot2 R software. All the tests were two-sided, and P< 0.05 was considered statistically significant.

## Results

### K-means algorithm identifies two metaClusters in PCa

**Figure 1:**
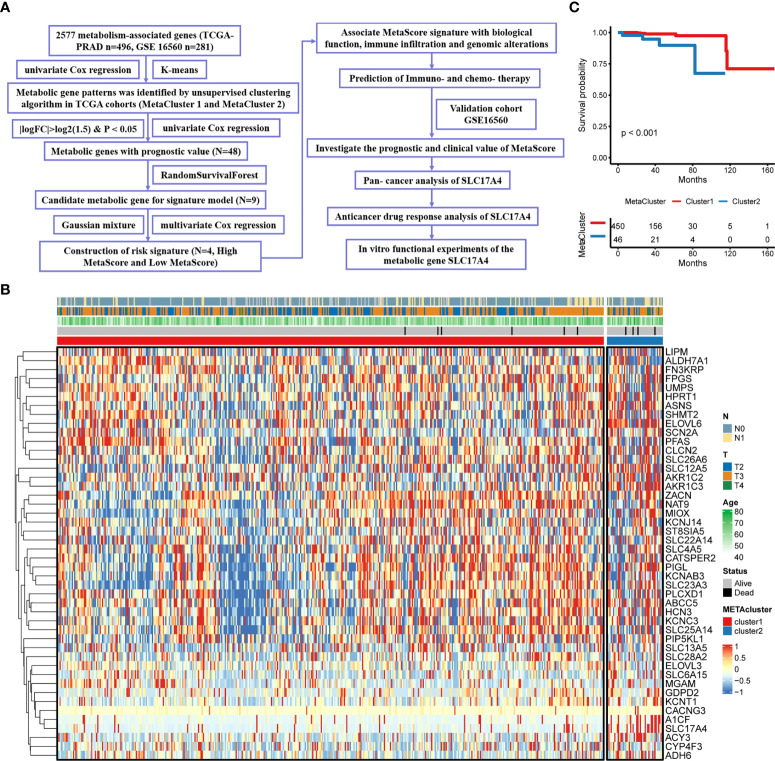
Characteristics of MetaCluster in PCa. **(A)** Flow chart of the study. **(B)** Heatmap of the candidate genes associated with MetaCluster. **(C)** Kaplan–Meier curves showing the correlation between MetaCluster and OS (log-rank test, P< 0.001).

Flowchart **Figure 1A** comprehensively described our study. In order to characterize metabolic heterogeneity within PCa, 46 candidate metabolic genes were confirmed for clustering using univariate cox regression. By conducting consensus clustering on the gene expression pattern of candidate genes, two resulting clusters were defined, MetaCluster 1 and MetaCluster 2. The heatmap for the expression of the 46 metabolic identified hub genes of the 496 patients is shown in **Figure 1B**. Notably, significant prognostic differences were observed between the two subclusters, with shorter OS for MetaCluster 2 than MetaCluster 1 (P < 0.001, **Figure 1C**). Furthermore, the correlation analyses between MetaCluster and the activity of 114 identified metabolic pathways were presented in **Figure S1** and **Table S1**. Results revealed distinct metabolic patterns between MetaCluster 1 and MetaCluster 2.

### The model constructed by multivariate cox regression analysis

**Figure 2:**
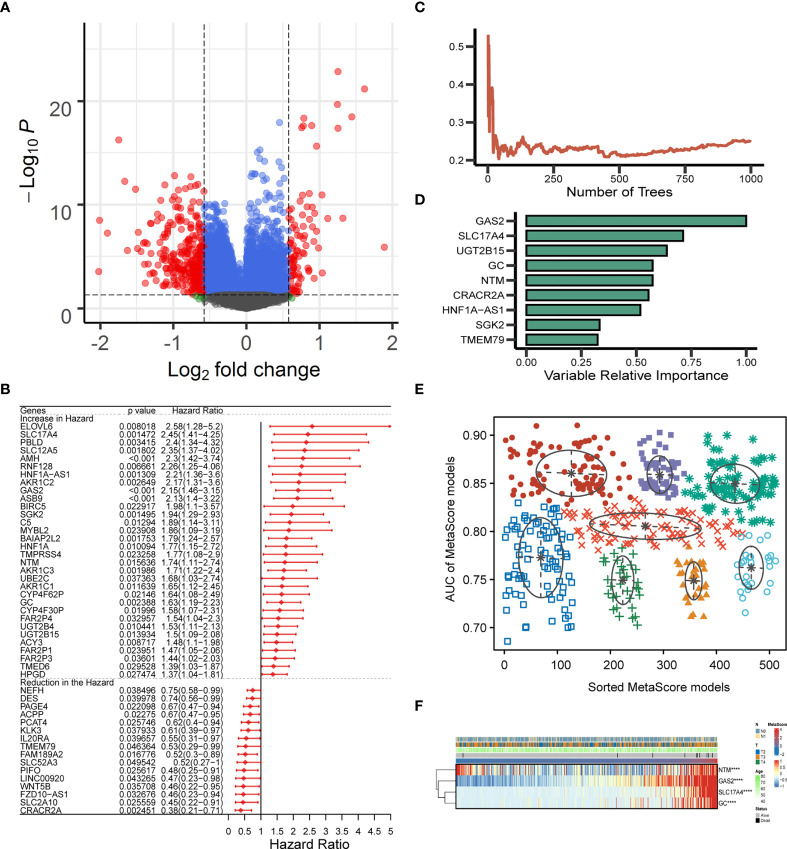
Establishment and verification of MetaScore signature. **(A)** The volcano plot of mRNA levels expression of metabolism genes. The abscissa is the log2 value of the screening condition, the ordinate is the log10 transformed p-value. The red-colored dots represent the DEGs (|logFC|> log2(1.5) and P< 0.05). **(B)** Univariate Cox analysis of 48 selected genes. **(C, D)** The error rate of the random trees and variable relative importance for the 9 metabolism-related genes. **(E)** The pattern of the logistic regression model is related to the AUC scores and is verified by a Gaussian mixture, including 8 clusters of 511 combinations. **(F)** The heat map revealed the relationship between the four-gene signature and distribution of MetaScore ****P < 0.0001.

The identified candidate metabolic genes (absolute (log fold change) > log2(1.5) & P < 0.05) were showed by Volcano plot (**Figure 2A**). After analyzing the selected gene with univariate cox regression, 48 prognostic genes were achieved: 32 increased in Hazard Ratio and 16 reduced in Hazard Ratio (**Figure 2B**). We constructed a prognostic model containing nine genes. The development of a random survival forest model and the importance of nine variables are exhibited in **Figures 2C, D** Subsequently, the Gaussian mixture model (GMM) combined with ROC curves was established to evaluate the predictive ability of the signatures by calculating the AUCs, the highest AUC as our model to predict the OS of PCa patients was selected from the eight clusters (**Figure 2E**). Hence, we finally established a prediction signature comprising four genes (NTM, GAS2, SLC17A4, GC), heatmap for the four-gene signature is shown in **Figure 2F**. MetaScore = 0.5154*GAS2 + 0.395*SLC17A4 + (- 0.1211*NTM) + 0.2939*GC.

**Figure 3:**
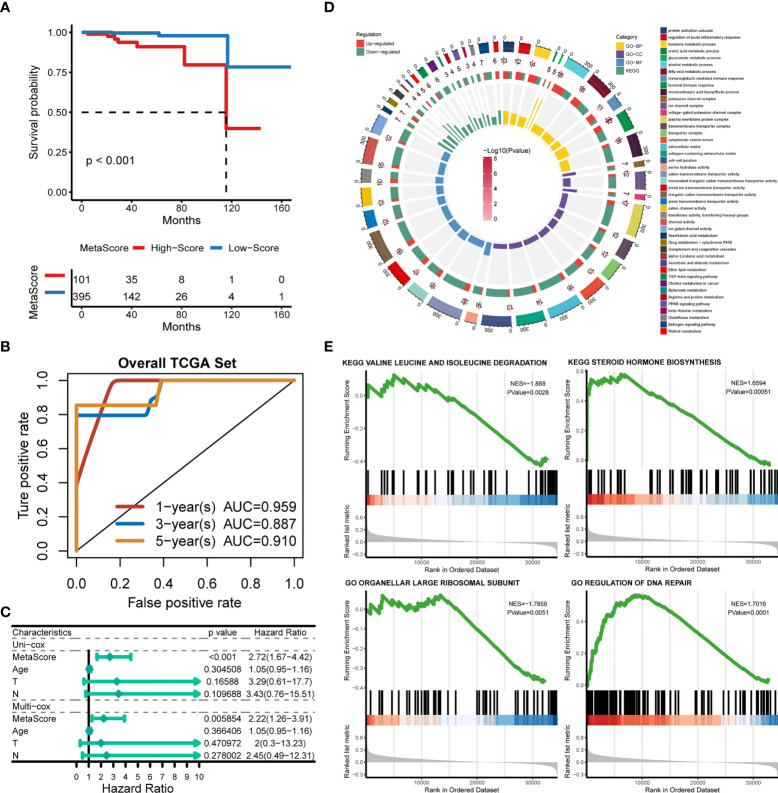
Functional annotation of low- and high-MetaSore groups. **(A)** Kaplan–Meier curves showing the correlation between MetaScore and OS (log-rank test, p < 0.001). **(B)** ROC curves exhibited the predictive capability of the MetaScore signature on the clinical value including 1-, 3- and 5-year. **(C)** Clinical variables related to OS by univariate cox and multivariate cox analysis in the TCGA-PRAD cohort. **(D)** GO and KEGG plots for enrichments based on the high- and low-MetaScore group. **(E)** GSEA plots for enrichments based on the high- and low-MetaScore group.

Patients were classified into the high- and low-MetaScore groups according to the best cutoff value of the metabolic score, termed as MetaScore, which was calculated by the four-gene signature. We then ranked the samples using MetaScore in the training cohort and internal validation cohort. The relevance between survival probability and MetaScore of patients was explored (**Figure 3A**). The survival analysis revealed that patients with low-MetaScore related to a better OS. What`s more, the predictive ability of MetaScore signature was validated in the GSE16560 cohort (**Figure S2**). The 1-, 3-, and 5-year ROC curves demonstrated a promising AUC of 0.959, 0.887 and 0.910, respectively (**Figure 3B**). The AUC suggested excellent clinical value in predicting the short- and long-term survival probability in PCa. Afterward, univariate and multivariate cox regression identified MetaScore as an OS-related factor (**Figure 3C**).

### Biological behaviors of the metabolic genes

**Figure 4:**
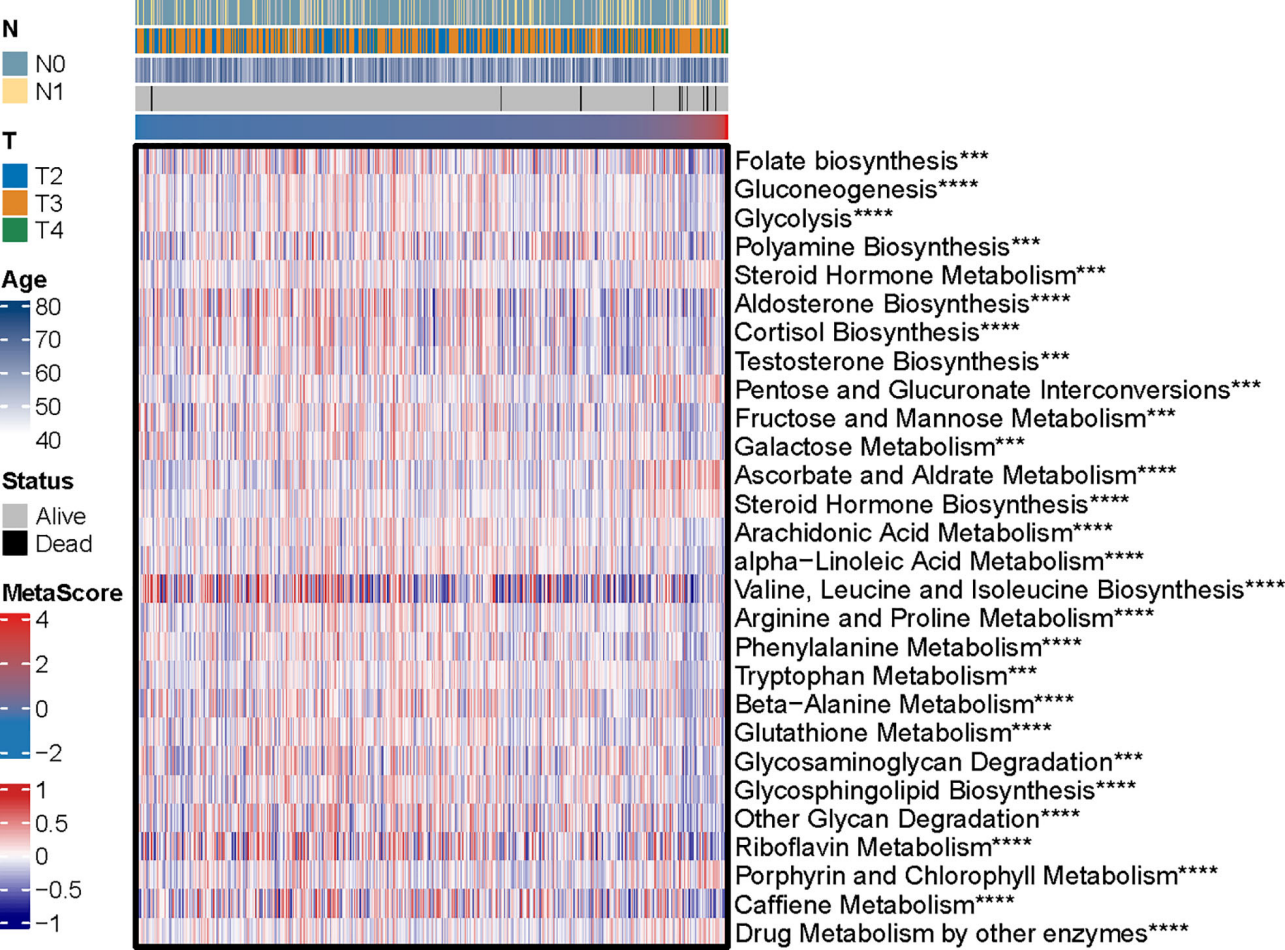
The heatmap was employed to visualize the most significant pathways among 114 identified metabolic-related pathways correlation to MetaScore. ***p < 0.001, ****P < 0.0001.

The potential functions and pathways in involved differentially expressed metabolic genes in PCa were determined using GSEA analysis. Fifteen metabolic-, immune-related signaling and tumorigenic pathways in KEGG, and 30 GO annotations were determined (**Figure 3D**). Enrich GO analyses revealed that the upregulation was mainly annotated to humoral immune response, cation transmembrane transporter activity, inorganic cation transmembrane transporter activity, alcohol metabolic process, fatty acid metabolic process, and monocarboxylic acid biosynthetic process (**Figure 3D** and **Table S2**). Enrich KEGG analyses demonstrated that the specific metabolic pathways were mainly gathered in arachidonic acid metabolism, drug metabolism-cytochrome P450, complement and coagulation cascades, estrogen signaling pathway, TGF-beta signaling pathway, choline metabolism in cancer, and retinol metabolism (**Figure 3D** and **Table S3**). Moreover, GSEA was also performed to determine the functional enrichments of each subtype (**Tables S4**, **S5**). We found that regulation of DNA repair and steroid hormone biosynthesis were activated, while organellar large ribosomal subunit and valine leucine and isoleucine degradation were relatively suppressed in it (**Figure 3E**). The association between MetaScore and the activity of 114 identified metabolic pathways was explored and the most significant pathways was presented (**Figure 4**)

### MetaScore is related to distinct profiling of genomic alterations

**Figure 5:**
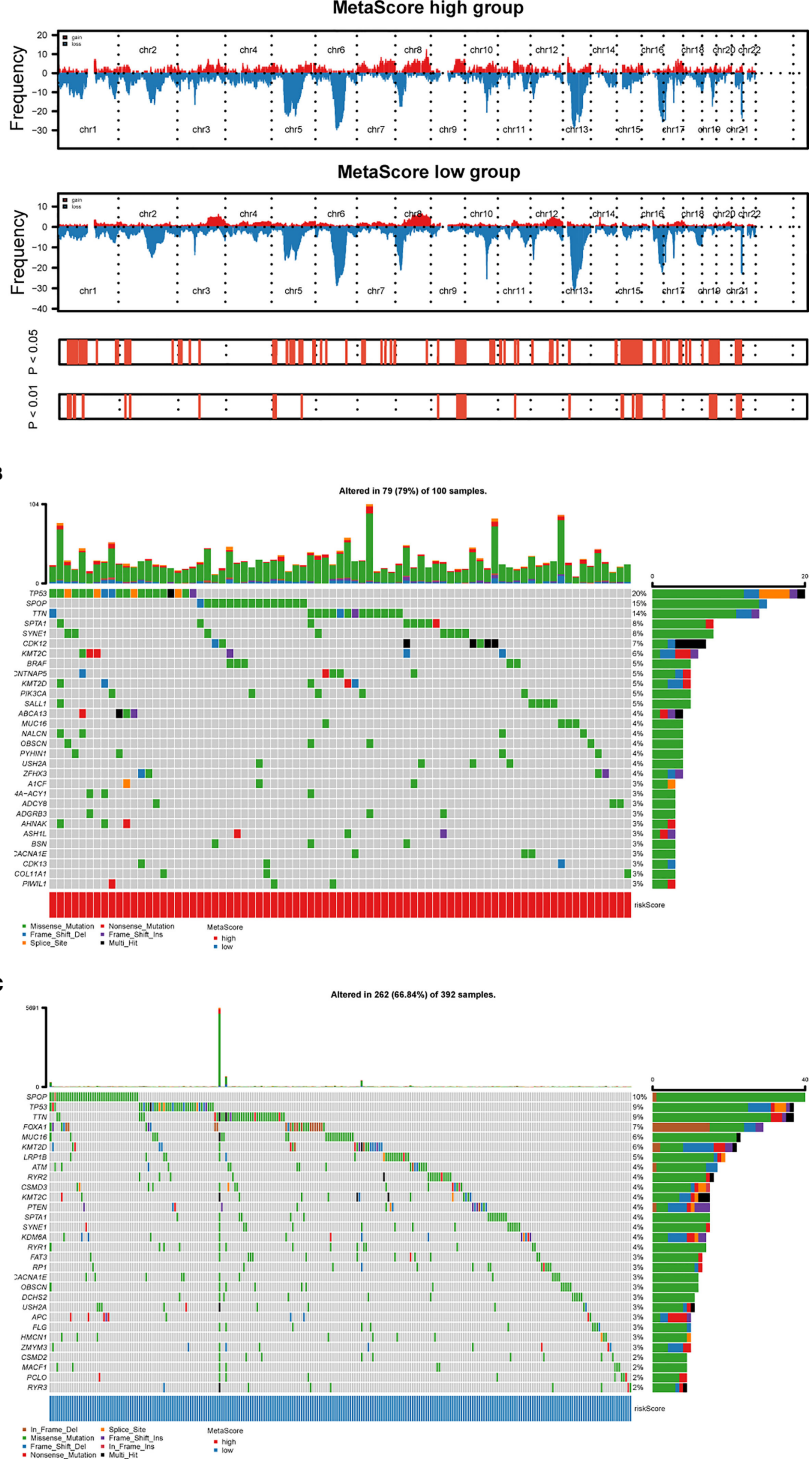
Distinct genomic pattern related to MetaScore. **(A)** Amplifications and deletions in PCa with high- and low-MetaScore. Chromosomal regions of peaks correspond to the relevant recurring focal amplification (red) and deletions (blue). **(B, C)** The overall somatic mutation profile with the highest frequency in high- and low-MetaScore groups. P< 0.05 indicates statistical significance.

To explore the relationship between MetaScore and genomic patterns in PCa, CNAs and somatic mutation analyses were performed. We next assembled copy number variation regions (CNVRs) by merging overlapping CNVs of the type (loss or gain) (**Figure 5A**). What`s more, analysis of somatic mutation patterns demonstrated a high incidence of mutations in TP53 (20%), SPOP (15%), TTN (14%), SPTA1 (8%), SYNE1 (8%), CDK12(7%) and KMT2C (6%) in the high MetaScore group (**Figure 5B**), while SPOP (10%), TP53 (9%), TTN (9%), FOXA1(7%), MUC16 (6%) and KMT2D (6%) presented higher-incidence mutations in the low MetaScore group (**Figure 5C**).

### Immune infiltration of metabolic subtypes in PCa

The immune cell infiltration in the tumor microenvironment of the TCGA-PRAD between high- and low-MetaScore groups was presented through a heatmap (**Figure S3**). Monocytes, Macrophages M0, Dendritic cells activated, CD56bright.natural.killer.cell, CD56dim.natural.killer.cell, Eosinophil, Immature.dendritic.cell Plasmacytoid.dendritic.cell, and Type.17.T.helper.cell, were enriched in the low-MetaScore group. B cells naive, Monocytic lineage, Endothelial cells, Activated.CD4.T.cell, Central.memory.CD8.T.cell, Type.2.T.helper.cell, and B cell were enriched in high-MetaScore group. What`s more, we also demonstrated the correlation between infiltration of immune cells and MetaScore with a heatmap (**Figure S4**). The expression of the immune checkpoint is the trigger of tumor-intrinsic immune escape, and the involved molecules include antigen-presenting cells, co-stimulators, co-inhibitors, receptors, ligands, cell adhesions, etc (36, 37). Therefore, we investigated the correlation between the immune checkpoint and MetaScore (**Figure S5**).

### The role of MetaScore in the prediction of immunotherapeutic benefits

**Figure 6:**
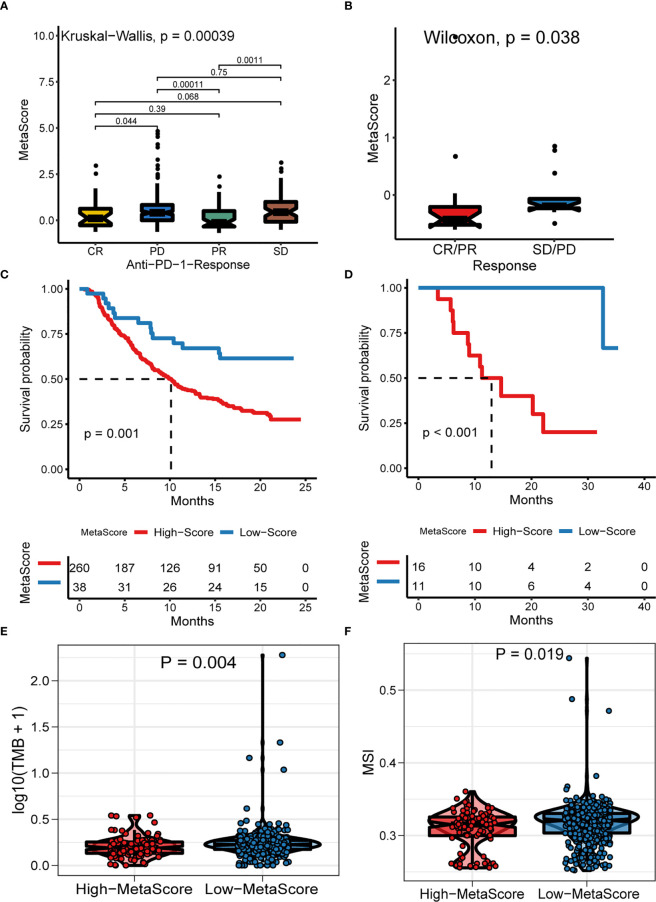
The Role of MetaScore in the prediction of immunotherapeutic benefits. MetaScore in groups with a different anti-PD-1 clinical response status (complete response [CR]/partial response [PR] and stable disease [SD]/progressive disease [PD]) in the IMvigor210 cohort **(A)** and GSE78220 cohort **(B)**. Kaplan-Meier curves for patients with high- and low-MetaScore in the IMvigor210 cohort **(C)** and GSE78220 cohort **(D)**. **(E)** The differences of TMB between MetaScore groups in the training set. **(F)** The differences of MSI between MetaScore groups in the training set. TMB, tumor mutation burden; MSI, microsatellite instability.

Emerging immune checkpoint blockade therapies blocking the programmed death 1 (PD-1) or its ligand PD-L1 molecules have exhibited satisfactory outcomes, with the potential to prevent the progress of advanced cancer. Therefore, we evaluated the utility of the MetaScore in estimating the therapeutic benefit in patients. For this purpose, the patients who adopted anti-PD-L1 immunotherapy in the IMvigor210 cohort were assigned high- and low-MetaScore groups according to our four-gene signature. Notably, MetaScore is related to objective response to anti-PD-L1 therapy in the IMvigor210 cohort (Kruskal-Wallis, p = 0.00039; **Figure 6A**). Patients with low MetaScore significantly outlived patients with high MetaScore in the IMvigor210 cohort (log-rank test, p = 0.001; **Figure 6C**). A similar outcome was observed in the GSE78220 cohort, which was also undergoing anti-PD-1 checkpoint inhibition therapy (Wilcoxon, p =0.0038, **Figure 6B**; log-rank test, p < 0.001, **Figure 6D**). Further, we verified the immunotherapeutic response in GSE35640 and GSE91061 cohorts, which received distinct immunotherapies (Wilcoxon, p =0.0016; **Figure S6A**; Wilcoxon, p =0.0024; **Figure S6B**). TMB and MSI were emerging biomarkers associated with immunotherapy response. Thus, the correlation between MetaScore and TMB/MSI in TCGA-PRAD was further investigated (**Figures 6E**, **F**). Collectively, these data demonstrated that MetaScore might serve as hazardous prognostic markers and predict immunotherapy response.

### Prognostic metaScore and sensitivity to chemotherapy

**Figure 7:**
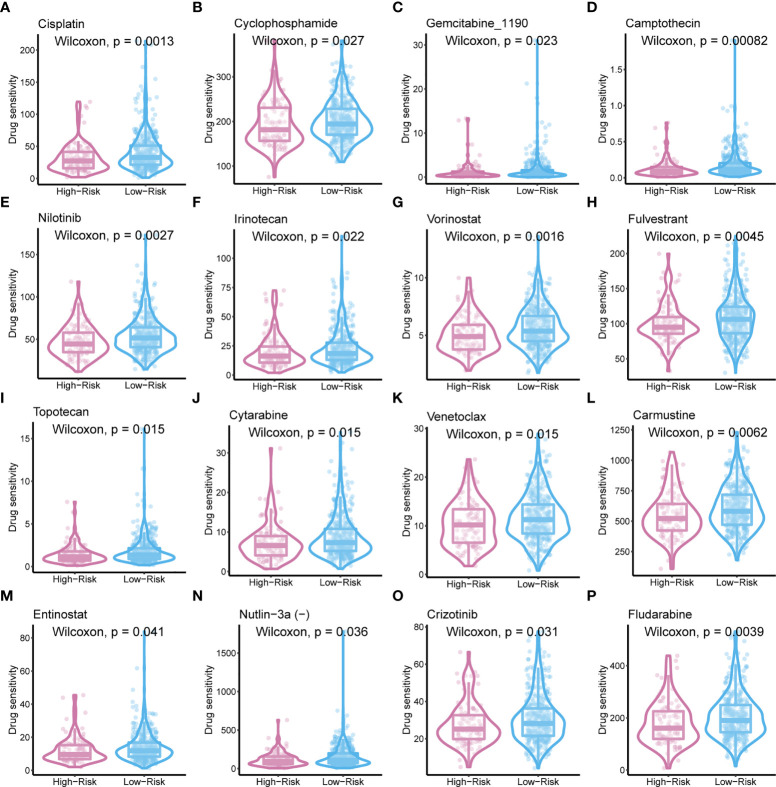
The sensitivity of 16 common chemotherapy drugs between MetaScore groups in PCa cells.

**Figure 8:**
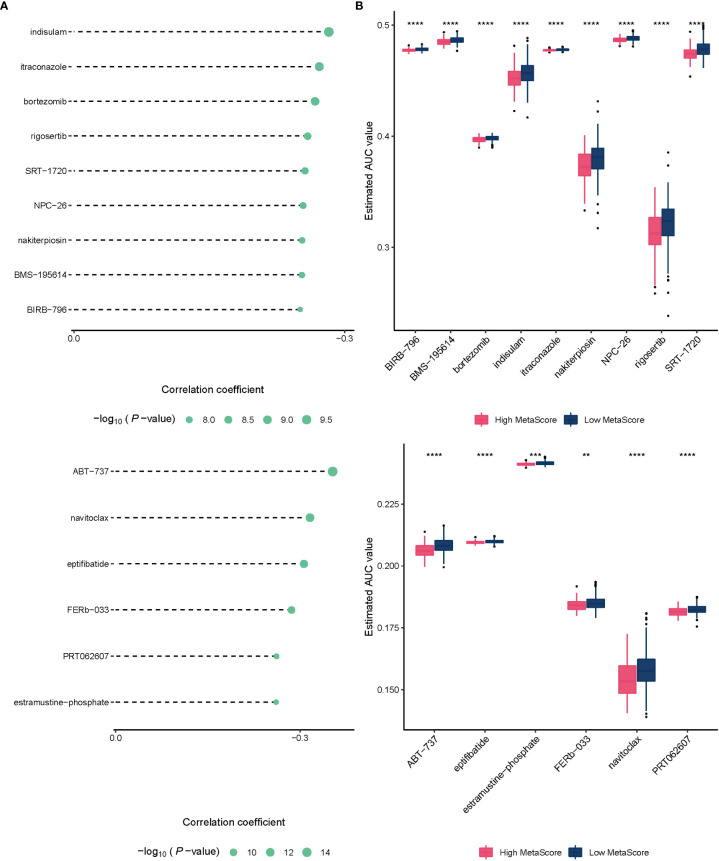
**(A)**The correlation coefficient of 15 chemotherapy drugs. **(B)** The estimated AUC value of 15 chemotherapy drugs between MetaScore groups. **p < 0.01, ***p < 0.001, ****P < 0.0001.

**Figure 9:**
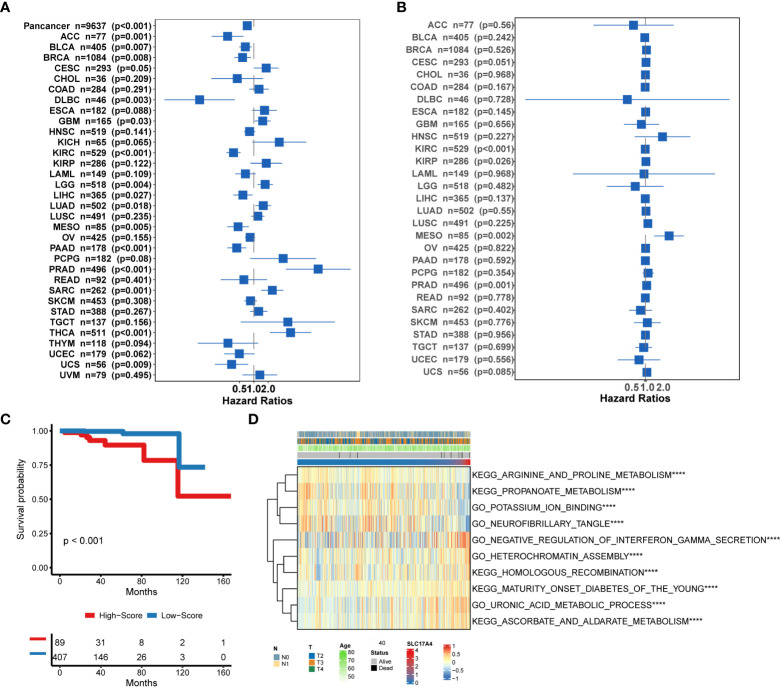
**(A)** Pan-cancer analysis of the 4-gene signature based on TCGA. **(B)** Correlation between the expression of SLC17A4 and overall survival in multiple tumor types based on TCGA. **(C)** Survival curves of OS between low-SLC17A4 patients and high-SLC17A4 based on TCGA-PRAD. **(D)**The differential in GO and Enrich analysis between low- and high-SLC17A4 groups. ****P < 0.0001.

To improve the therapeutic outcomes of PCa patients, we further investigated the correlation between our MetaScore and the predicting sensitivity to 16 common chemotherapy drugs (**Figure 7**). The analysis revealed that increased MetaScore was related to increased drug sensitivity of cancer cells to Cisplatin, Cyclophosphamide, Gemcitabine, Camptothecin, Irinotecan, Vorinostat, Fulvestrant, Topotecan, Cytarabine, Venetoclax, Carmustine, Entinostat, Nutlin-3a, Crizotinib, Fludarabine, Nilotinib. The correlation coefficient and corresponding estimated AUC value of another 15 chemotherapy drugs were shown in **Figure 8**. What`s more, in order to evaluate the broad applicability of the four-gene signature, pan-cancer analysis (33 tumors) was conducted based on TCGA and the Harzard Ratios suggested that the four-gene signature can be an increased risk predictor for 7 tumors (**Figure 9A**).

### Bioinformatic analysis of molecular mechanisms underlying SLC17A4

**Figure 10:**
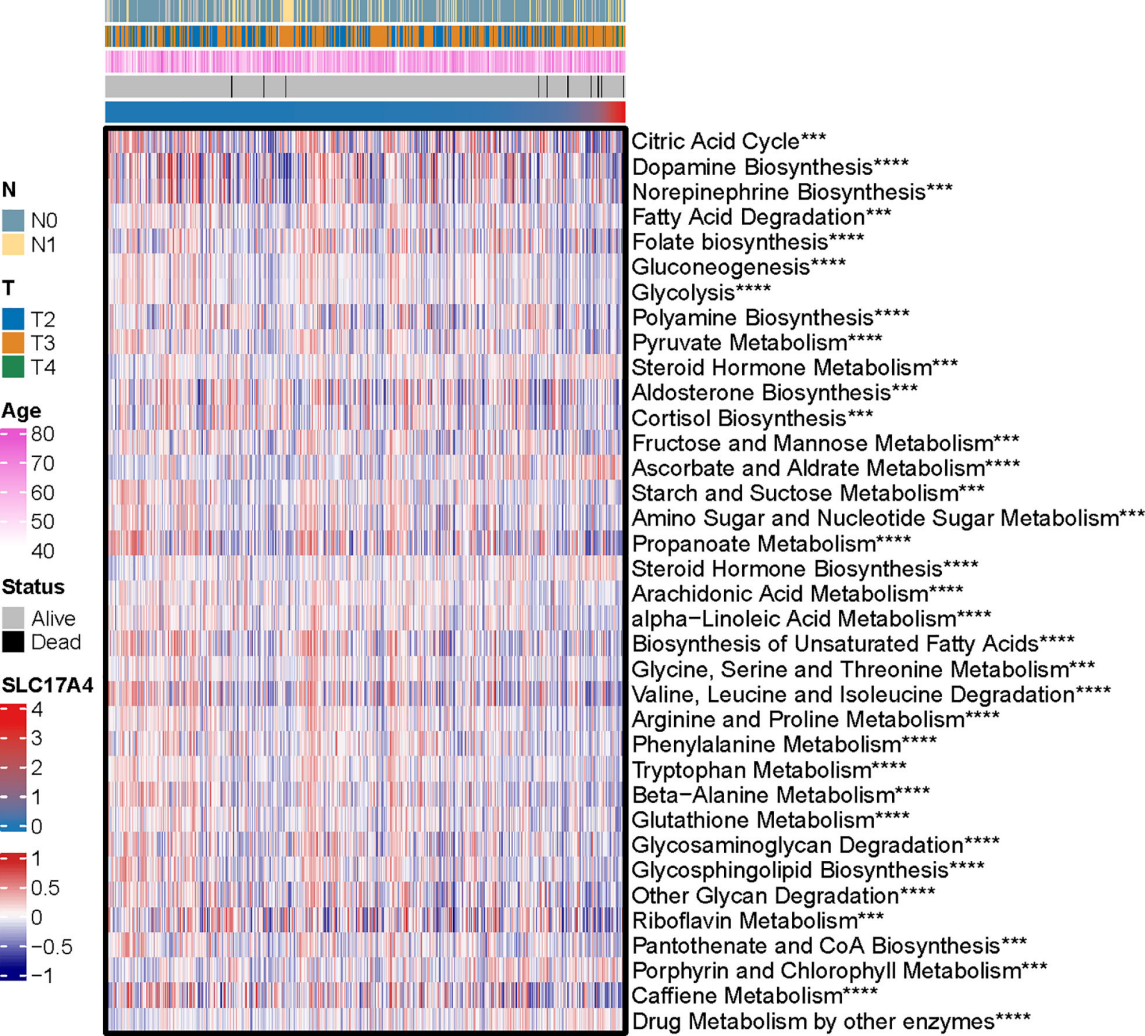
The correlation analysis between the 114 identified metabolic-related pathways and SLC17A4 (the most significant pathways was presented). ***p < 0.001, ****P < 0.0001.

**Figure 11:**
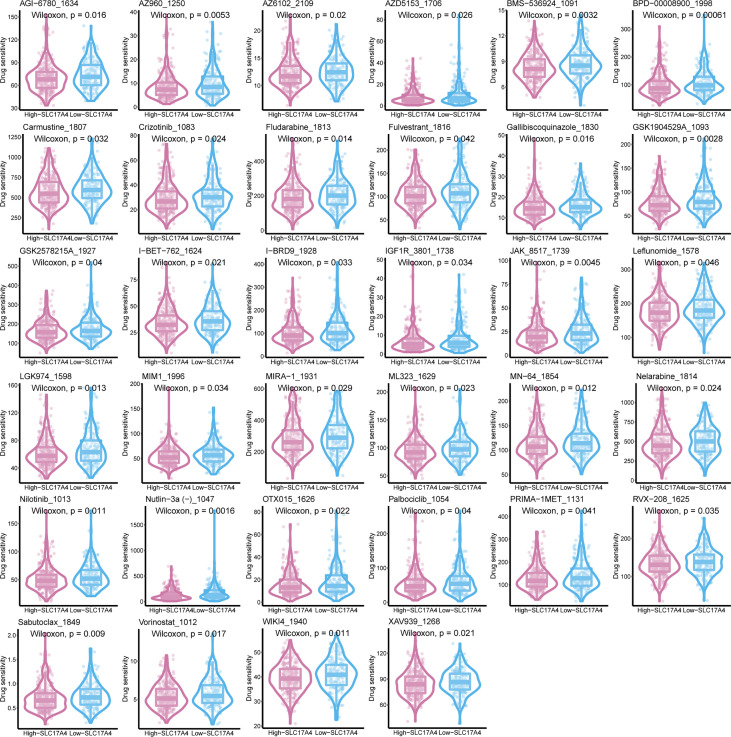
The IC50 of 38 chemotherapy drugs in PCa cells was estimated in SLC17A4 groups utilizing the GDSC database.

Inspired by the satisfactory prognosis predicting ability of the four-gene model in the training and validation cohort, we further applied bioinformatic analyses to explore the potential underlying mechanisms of SLC17A4. To our knowledge, the role of SLC17A4 in PCa has not been explored. The correlation between the expression of SLC17A4 and prognosis was evaluated in 33 tumor types (**Figure 9B**). Based on the cox analysis, we suggested that SLC17A4 might be an oncogene for the 3 evaluated types of tumor. Kaplan-Meier analysis indicated that low expression of SLC17A4 is associated with better survival in PCa (P < 0.01, **Figure 9C**). Furthermore, GSVA revealed that three GO category negative regulation of interferon gamma secretion, uronic acid metabolic process, heterochromatin assembly were highly enriched in the high-SLC17A4 group, three KEGG category ascorbate and aldarate metabolism, maturity onset diabetes of the young, homologous recombination were mainly gathered in the high-SLC17A4 group (**Figure 9D**). The correlation analyses between SLC17A4 expression and the most significant identified metabolic pathways among 114 are shown in **Figure 10**. To determine the potential therapeutic drugs in high- and low-SLC17A4 PCa patients, the IC50 of 34 drugs in PCa cells was estimated utilizing the GDSC database. Remarkably, the drug sensitivity (IC50) of 34 chemotherapy compounds was significantly lower in the high-SLC17A4 group as compared to the low-SLC17A4 group, which revealed that the patients with high-SLC17A4 could be more beneficial to the application of these drugs (**Figure 11**).

### SLC17A4 regulates the invasion, viability, and proliferation of PCa

**Figure 12:**
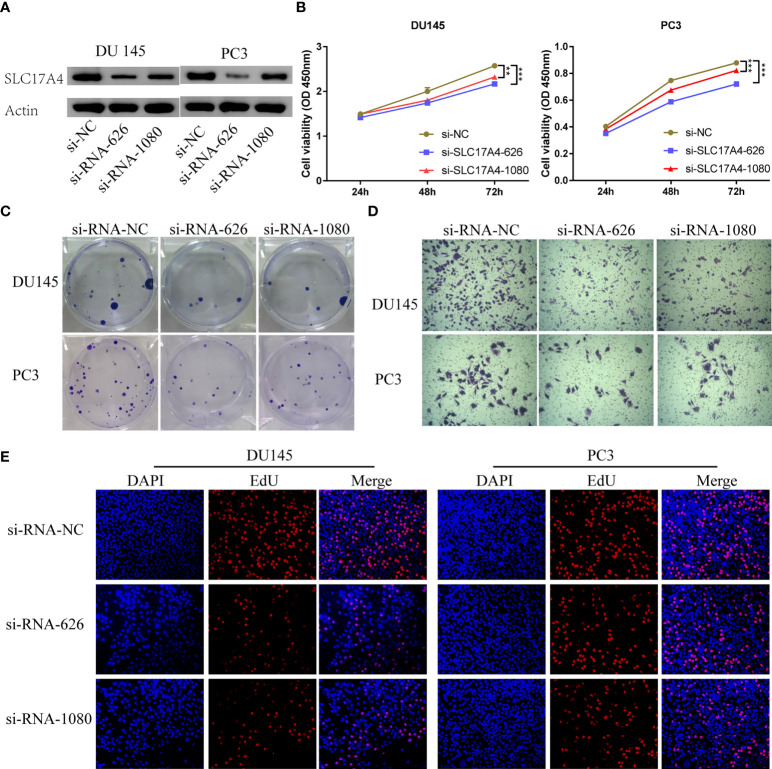
**(A)** Measurement of siRNA transfection efficiency in DU145 and PC3 cells at the protein level. **(B)** CCK-8 assays revealed that silence of SLC17A4 suppressed the proliferation of DU145 and PC3 cells. **(C)** Colony formation assay of DU145 and PC3 cells after the knockdown of SLC17A4. **(D)** Knock-down of SLC17A4 affected the invasion ability of DU145 cells and PC3 cells (Crystal Violet Staining). **(E)** EdU assay of PC3 and DU145 cells after the knockdown of SLC17A4 (siRNA-NC: siRNA negative control). **p < 0.01, ***p < 0.001.

Various *in vitro* experiments were performed to validate the pathogenic role of SLC17A4 in PCa cells. Transfection in DU145 and PC3 cells was performed using three siRNAs to prohibit the expression of SLC17A4, in which si-RNA-626 and si-RNA-1080 exhibited relatively high efficiency (Figure S7). Western blot was employed to verify the silence of SLC17A4 by siRNA (**Figure 12A**). The CCK8 assay interpreted that the cell proliferation ability is inhibited by silencing SLC17A4 (**Figure 12B**). The colony formation experiment revealed that the inhibition of SLC17A4 remarkably reduced the colony number in DU145 cell line and PC3 cell line (**Figure 12C**). Dramatically, the dysfunction of SLC17A4 inhibited the invasion ability of DU145 and PC3 cells (**Figures 12D**, **S8A**). What`s more, the EdU assay indicated that the proliferation ability of PCa cells was inhibited by the silence of SLC17A4 (**Figures 12E**, **S8B**). Therefore, the prognostic gene SLC17A4 was associated with the proliferation and invasion of PCa cells and may be a potential therapeutic target for PCa.

## Discussion

Metabolic reprogramming has emerged as a prominent hallmark of tumors (38). Tumor cells alter their dominant oxidative phosphorylation ATP-producing procedure to aerobic glycolysis even if there is sufficient oxygen (described as the Warburg effect) (39). The metabolic characteristics of cancer cells might influence varying cells in the TME. Among them are tumor-associated fibroblasts, endothelial cells and immunocytes, which eventually facilitate the invasion, proliferation and no response to antitumor therapy of cancer cells (40). A study reported that upregulation of glycolytic metabolism might accelerate prostate cancer progression and radioresistance *via* circular RNA (41). Thereby, targeting the metabolic state of cancers with drugs would be a promising therapeutic approach for better outcomes. In this study, PCa patients were stratified into MetaCluster 1 and MetaCluster 2 according to metabolic genes to investigate the correlation between tumor metabolic profile and tumor phenotype. Differentially expressed metabolic-associated pathways were verified between the two MetaClusters. Subsequently, to further investigate the correlation between the cluster model and cancer progression pattern, we establish a scoring system, MetaScore, to qualify the gene model. In summary, we focus on analyzing the possible biological behavior of metabolism-associated genes in the prognosis and development of PCa by bioinformatics analysis and functional cell assays.

Integrating multiple biomarkers into one aggregate signature *via* bioinformatics might enhance the prediction compared with a single biomarker (42, 43). Here, we applied a multi-step bioinformatics analysis to establish a metabolic genes model to predict OS in PCa patients. We identified four metabolism-associated genes (GAS2, SLC17A4, NTM, and GC) related to OS in PCa. Among the four genes, the NTM gene showed negative coefficients, and the expression was upregulated in promising OS patients. What`s more, the expression of GAS2, SLC17A4, and GC was increased in patients with unfavorable outcomes, which have positive coefficients. Studies showed that some of the 4 genes are involved in tumors, including PCa. It has been reported that the 1,25(OH)2 D/25(OH)D (metabolites of GC) molar proportion was related to a reduced risk of high aggressive PCa in African-American men (44), vitamin D binding protein (the protein encoded by GC) regulates the correlation between total 25(OH)D expressions and risk of advanced and fatal PCa (45). However, the study interpreted that NTM may promote biochemical recurrence of PCa after radical prostatectomy *via* affecting regulatory T cells and M2 macrophages (46) and decreased expression of NTM in transformants is associated with hypermethylation close to the transcription start point in arsenic- or cadmium-transformed malignant prostate epithelial cells (47).

SLC17A4 is an organic anion transporter (belonging to the solute carrier 17 families) that is particularly maintained phosphate homeostasis. The study reported that phosphate transporters gene SLC17A4 is linked to Ca and P metabolism and homeostasis in pig models (48). Interestingly, a metabolizing enzyme colocalized with the SLC17A4 gene is closely related to thyroid hormone pathway, insulin signaling, and glucose metabolism (49, 50). Our analysis revealed that the upregulation of SLC17A4 increases MetaScore and is associated with poor prognosis in PCa patients. Our functional experiments further reveal that human SLC17A4 is capable of promoting progression and invasion in PCa cells (**Figure 12**). Herein, for the first time, we recognized the roles of SLC17A4 in the development and progression of PCa. Further exploration is needed to verify the biological function and underlying mechanism of SLC174A in PCa biogenesis and progression. Further characterization of molecules from the signature will supply novel insights into the tumor etiology and may reveal potential metabolic therapeutic targets.

The tumor cell metabolism affects TME and immune infiltration patterns, thereby altering the efficiency of checkpoint-based immunotherapy. The metabolic status is different between normal tissue and PCa, so it provides a new way to identify cancers through metabolic differences. It has been interpreted that PCa cells show high consumption of glucose during the metastatic stage (12, 13), and PCa patients with highly glycolytic metabolism may promote tumor progression and aggressiveness (14). The accumulation of lactic acid, the metabolic product of glycolysis, in the extracellular matrix is conducive to the acidic TME and further influences immune cell infiltration. A study demonstrated that acidic TME might restrict T cell-mediated immunity and promote hyporesponsiveness of immune cells (51). Consistently, immunocytes including B cells naive, Monocytic lineage, Endothelial cells, Activated.CD4.T.cell, Central.memory.CD8.T.cell, Type.2.T.helper.cell, and B cell in the high-MetaScore group presented more disordered than low-MetaScore group (**Figure S3**).

Immune checkpoint inhibitors have presented promising outcomes in treating patients with various cancers, providing new frontiers in cancer treatment strategies (52, 53). PCa has been stratified into an immune-desert pattern and is moderately responsive to immunotherapy (54, 55). Therefore, only partial and specific patients might benefit from the immunotherapy. Although biomarkers have been extensively explored to predict PCa prognosis, metabolic signatures for predicting the response of immuno-/chemo-therapy have not been developed. In the present study, we developed a novel system according to metabolic genes to predict the efficacy of immunotherapy. TMB and MSI were emerging biomarkers associated with immunotherapy response (56). Patients with higher TMB and MSI may benefit more from the treatment of immunotherapy (57), which is consistent with our findings (**Figure 6**).

Given the complexity and diversity of PCa cell lines, the selected PC3 and DU145 cell lines (less differentiated and androgen-independent) may not represent the full spectrum of the disease, a wider variety of PCa cell lines should be used in subsequent studies.

Our study is the first to comprehensively elucidate the chemo-/immuno-therapy response of PCa patients based on MetaGene-signature. A recent study reported only chemotherapy response based on MetaGene-signature but lacked immunotherapy response results (58). Interestingly, studies have shown that signature based on MetaGenes are associated with PCa recurrence undergoing radical prostatectomy (59). Two other researches also revealed that the model established by MetaGenes can predict the prognosis of PCa, which is consistent with our study (60, 61). In summary, we conducted an integrated analysis to develop a metabolism-based four-gene signature for predicting the OS and chemo-/immuno-therapy response of PCa patients. This study investigated the expression patterns, prognostic value, and potential mechanisms of metabolic genes in PCa. Future prospective clinical trials are required to assess the clinical utility of this signature.

## Data availability statement

The datasets presented in this study can be found in online repositories. The names of the repository/repositories and accession number(s) can be found in the article/**Supplementary Material**.

## Author contributions

JG and HL performed database analysis and drafted the manuscript. NZ and RJP contributed to all the designs and revise of the study. YQT, SYL, HZ, ZYD and ZYW, made contributions to the drafting of the manuscript. All authors reviewed and approved the final draft of the study.

## Funding

This work was supported by National Natural Science Foundation of China (No. 81901268) and The Science and Technology Innovation Program of Hunan Province (2020RC2065), the Youth Natural Science Foundation of Hunan Province (2021JJ40321).

## Acknowledgments

JG (CSC NO.201906370030) was supported by a scholarship from the China Scholarship Council Program. Thanks to Shan Jiang, Tianran Zhang and Zhen Yuan for their support and help.

## Conflict of interest

The authors declare that the research was conducted in the absence of any commercial or financial relationships that could be construed as a potential conflict of interest.

## Publisher’s note

All claims expressed in this article are solely those of the authors and do not necessarily represent those of their affiliated organizations, or those of the publisher, the editors and the reviewers. Any product that may be evaluated in this article, or claim that may be made by its manufacturer, is not guaranteed or endorsed by the publisher.
