# Supplementary material for: A prognostic signature consisting of metabolism-related genes and SLC17A4 serves as a potential biomarker of immunotherapeutic prediction in prostate cancer
Source: Front Immunol. 2022 Oct 17;13:982628. doi: 10.3389/fimmu.2022.982628 (PMC9620963; doi:10.3389/fimmu.2022.982628)
Supplement: Supplementary file 1 [file DataSheet_1.doc]

Fig S1. The correlation analyses between MetaCluster and the activity of 114 identified metabolic pathways. *p < 0.05, **p < 0.01, ***p < 0.001, ****P < 0.0001.

Fig S2. Low MetaScore is correlated with better overall survival validated in GSE16560 cohort (log-rank test, P = 0.016).

Fig S3. The abundance of immune cells in high- and low-Metascore groups, which included four categories: CIBERSORT, ESTIMATE, MCPcounter, ssGSEA and TIMER. *p < 0.05, **p < 0.01, ***p < 0.001, ****P < 0.0001.

Fig S4. The correlation between infiltration of immune cells and MetaScore. *p < 0.05, **p < 0.01, ***p < 0.001, ****P < 0.0001.

Fig S5. The expression pattern of seven categories of immune checkpoints in high- and low-MetaScore groups. *p < 0.05, **p < 0.01, ***p < 0.001, ****P < 0.0001.

A B

Fig S6. Validation of immunotherapeutic response in GSE35640 cohort (A) and GSE91061 cohort (B)


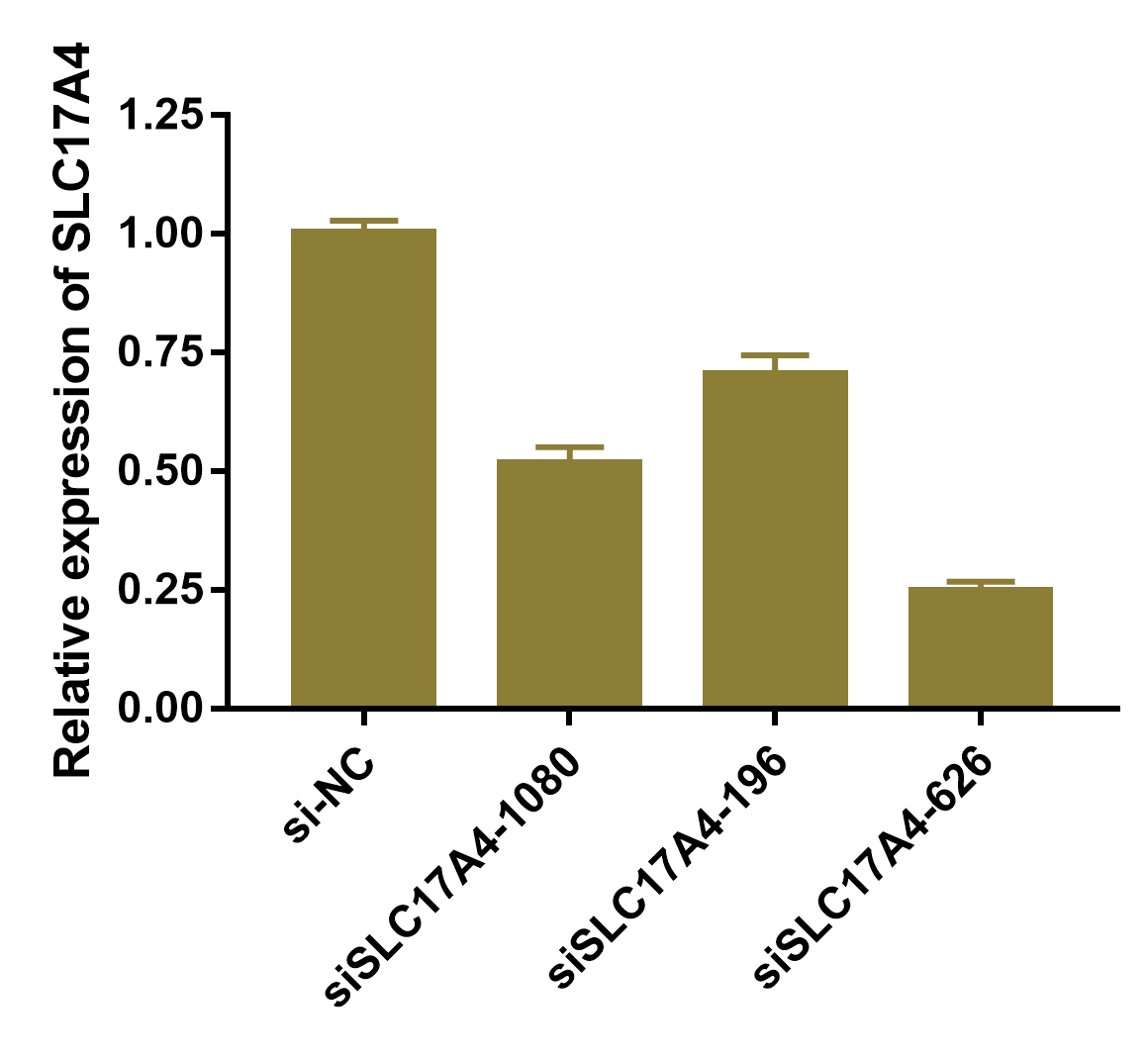


Fig S7. Relative expression of SLC17A4 after transfection in PC cells

A B


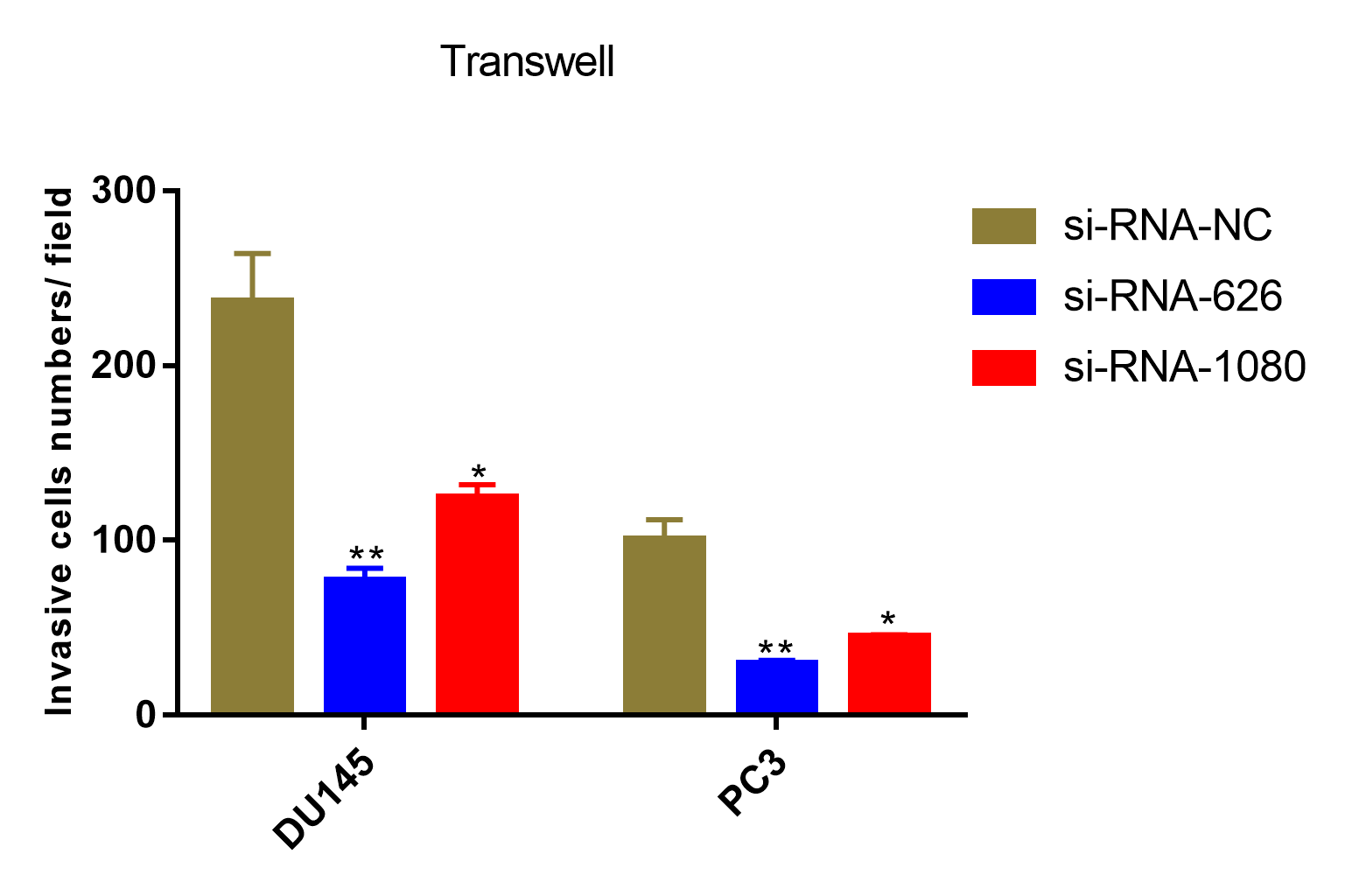

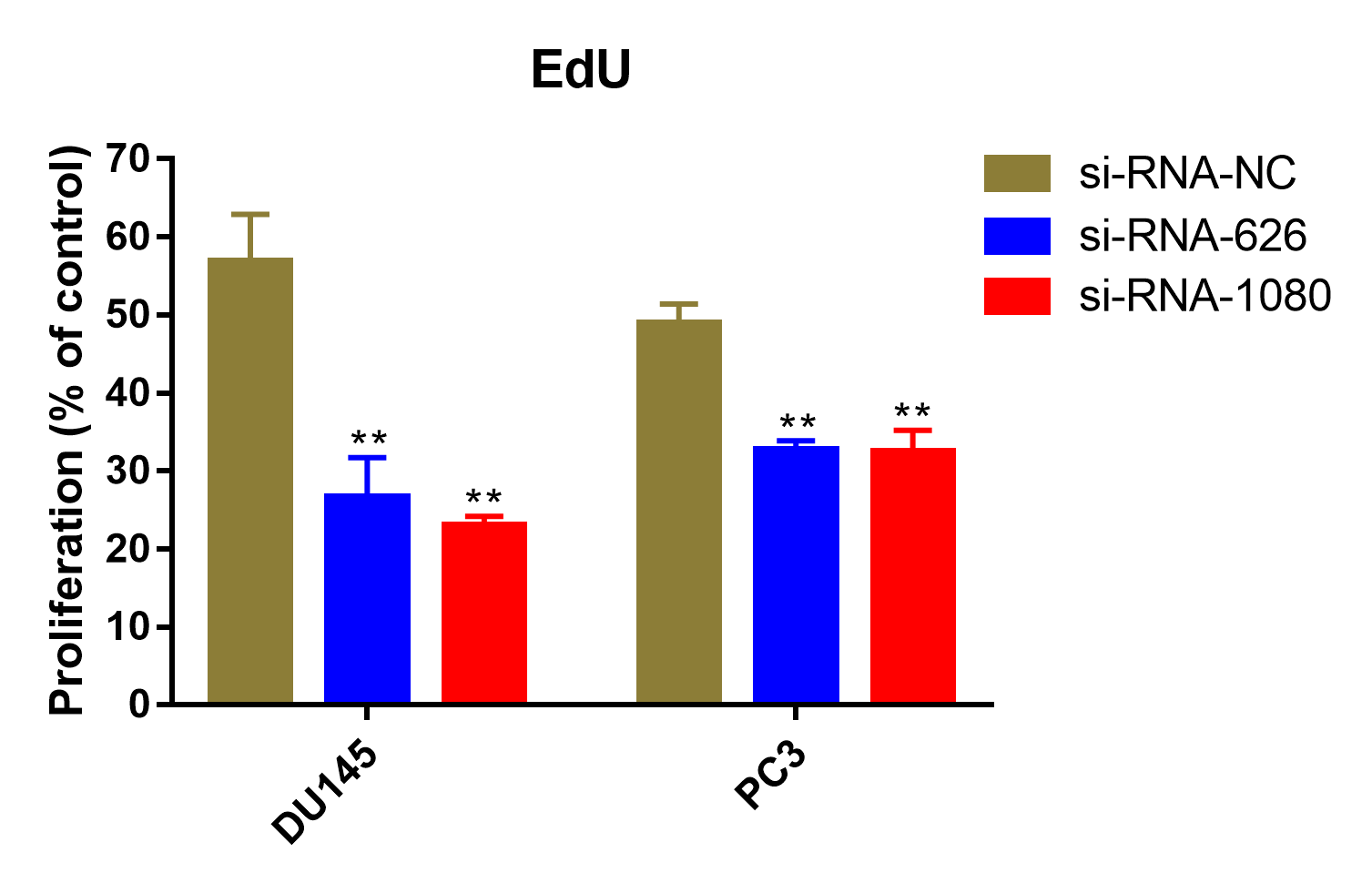


Fig S8. (A) Transwell assay of SLC17A4 knock-down DU145 cells and PC3 cells. (B) EdU assay of PC3 and DU145 cells after the knockdown of SLC17A4 (siRNA-NC: siRNA negative control) *p < 0.05, **p < 0.01, ***p < 0.001, ****P < 0.0001.
